# Integration and publication of heterogeneous text-mined relationships on the Semantic Web

**DOI:** 10.1186/2041-1480-2-S2-S10

**Published:** 2011-05-17

**Authors:** Adrien Coulet, Yael Garten, Michel Dumontier, Russ B Altman, Mark A Musen, Nigam H Shah

**Affiliations:** 1 LORIA – INRIA Nancy – Grand-Est, Campus Scientifique - BP 239 - 54506 Vandoeuvre-lès-Nancy Cedex, France; 2 Department of Medicine, 300 Pasteur Drive, Mail Code 5110, Stanford University, Stanford, CA, 94305, USA; 3 Department of Genetics, Mail Code 5120, Stanford University, Stanford, CA, 94305, USA; 4 Department of Biology, Carleton University, 1125 Colonel By Drive, Ottawa, ON, Canada, K1S5B6; 5 Department of Bioengineering, 318 Campus Drive, Mail Code 5444, Stanford University, Stanford, CA, 94305, USA

## Abstract

**Background:**

Advances in Natural Language Processing (NLP) techniques enable the extraction of fine-grained relationships mentioned in biomedical text. The variability and the complexity of natural language in expressing similar relationships causes the extracted relationships to be highly heterogeneous, which makes the construction of knowledge bases difficult and poses a challenge in using these for data mining or question answering.

**Results:**

We report on the semi-automatic construction of the PHARE relationship ontology (the PHArmacogenomic RElationships Ontology) consisting of 200 curated relations from over 40,000 heterogeneous relationships extracted via text-mining. These heterogeneous relations are then mapped to the PHARE ontology using synonyms, entity descriptions and hierarchies of entities and roles. Once mapped, relationships can be normalized and compared using the structure of the ontology to identify relationships that have similar semantics but different syntax. We compare and contrast the manual procedure with a fully automated approach using WordNet to quantify the degree of integration enabled by iterative curation and refinement of the PHARE ontology. The result of such integration is a repository of normalized biomedical relationships, named PHARE-KB, which can be queried using Semantic Web technologies such as SPARQL and can be visualized in the form of a biological network.

**Conclusions:**

The PHARE ontology serves as a common semantic framework to integrate more than 40,000 relationships pertinent to pharmacogenomics. The PHARE ontology forms the foundation of a knowledge base named PHARE-KB. Once populated with relationships, PHARE-KB (*i*) can be visualized in the form of a biological network to guide human tasks such as database curation and (*ii*) can be queried programmatically to guide bioinformatics applications such as the prediction of molecular interactions. PHARE is available at http://purl.bioontology.org/ontology/PHARE.

## Background

A large amount of biomedical knowledge is in the form of text embedded in published articles, clinical files or biomedical public databases. In order to construct computable knowledge bases from these sources, there is a great interest in capturing and formalizing this knowledge. The capture of relationships between biological entities is of particular interest since such relationships represent elementary and reusable knowledge units—often called “nano-publications” [1].

Our work is motivated by the need for automated approaches capturing and formalizing knowledge extracted from the literature via manual or computational approaches. Consider for example, that five curators at the Pharmacogenomics Knowledge Base (PharmGKB) manually browse the pharmacogenomics (PGx) literature to curate relationships relevant for storage in the PharmGKB [2]. The result of this curation process is a high quality database queried by clinicians and bioinformaticians. Nevertheless this manual curation process is not sustainable considering the growth of the scientific literature in this domain [3]. Automatic approaches using Natural Language Processing (NLP) are therefore increasingly utilized [4].

The simplest methods to capture relationships rely on co-occurrence of two entities to derive a relation between them. For example, in the sentence *“Our study shows that warfarin inhibits the expression of VKORC1”* a drug, *warfarin*, and a gene, *VKORC1*, can be recognized using simple lexicons. The co-occurrence of these two entities in one or more sentences is used to derive a relation of the form (*warfarin*, *VKORC1*)*.*

One key limitation of the co-occurrence based approach is identification of false positive connections. For example the sentence *“Warfarin inhibits the expression of VKORC1 while sulfamethoxazole inhibits the expression of CYP2C9”* would provide co-occurrence counts towards four relationships including the relationships (*warfarin*, *VKORC1*) and (*warfarin*, *CYP2C9*); only one of which is true. A second limitation is the coarse granularity of the identified relationships. Considering the previous example, the mentioned relationship links *warfarin* and *theexpression of VKORC1*, and not *VKORC1* per se. We consider this distinction of importance since *VKORC1* and *expression of VKORC1* refer to a gene and a phenotype respectively—two very distinct entities. Despite these limitations, co-occurrence is successfully used to generate networks including protein-protein interaction networks, gene-disease networks and regulatory gene expression networks [5,6]. Most of these networks are hard to compute on since their representation format does not support queries with typed relationships and the semantics associated with the nodes and edges differ in every network.

**Figure 1:**
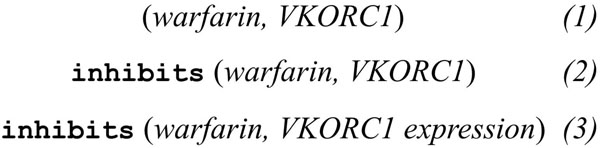
**Coarse to fine-grained relationships**. Coarse to fine-grained relationships identified in the sentence *“Our study shows that warfarin inhibits the expression of VKORC1”*. Relationships are mainly of three forms: (*1*) non-typed relationships composed of two atomic entities; (*2*) typed relationships between atomic entities; (*3*) typed relationships between atomic or composite entities.

Other NLP approaches can identify typed relationships and recognize entities that can either be the whole or a part of a subject and an object [7-9]. For example processing the previous sentence can identify the following relationship *inhibits*(*warfarin*, *the expression of VKORC1*) — that can also be represented as *inhibits* (*warfarin*, *VKORC1 expression*). Figure 1 shows three levels of granularity commonly encountered in text-mined relationships. Fine-grained relationships can be identified via syntactic parsing of sentences, which generates structures such as Parse Trees or Dependency Graphs (DG) [10]. In previous work, we presented a method based on syntactic parsing and DG exploration to extract fine-grained PGx relationships [11]. Given the variation in natural language, it is difficult to normalize the fine-grained and typed relationships extracted by this method. In this paper, we report on the construction of a relationship ontology and describe its use for integrating and publishing text-mined relationships on the Semantic Web. The relationships captured as instances of the PHARE ontology can be queried using Semantic Web technologies such as SPARQL and can be visualized in the form of a biological network. Semantics associated with relationships declared in PHARE-KB allow the text-extracted relationships to be consumed both by humans (for example, to guide curation) as well as by machines (for example, to guide computational prediction of molecular interactions).

## Methods

In previous work, we described the extraction of over 40,000 raw relationships in the domain of pharmacogenomics from MEDLINE abstracts [11]. In following sections we briefly summarize this extraction process and then describe how we use the PHARE ontology we have created to normalize and integrate these relationships.

### Relationships and PGx relationships

We define a relationship as a binary relation R (*a*, *b*), where *a*, and *b* are *subjects* and *objects* related by a relationship of type R. In PGx relationships *a* and *b* can be instances of a gene (*e.g.*, *VKORC1 gene*), drug (*e.g.*, *warfarin*), or phenotype (*e.g.*, *clotting disorder*). We note that *a* and *b* can also be entities that are related to genes (*e.g.*, VKORC1 expression), drugs (*e.g.*, warfarin dose) or phenotypes (*e.g.*, clotting disorder treatment). R is a type of relation described by words such as “inhibits”, “transports”, or “treats” and their synonyms.

**Figure 2:**
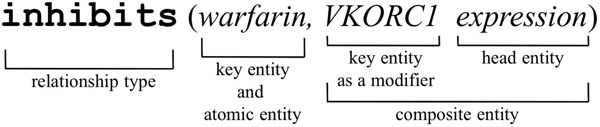
**Components of relationships.** A relationship has three components: relationship type, subject (here limited to a key entity), and object (here a composite entity which uses key entity as a modifier).

The three *key entities* in PGx (genes, drugs, and phenotypes) can be either direct targets for relation extraction, or indicators of latent PGx knowledge, as they modify other entities to create a second set of entities necessary to precisely describe PGx relationships. We refer to these modified entities as *composite entities* in contrast with the key entities. These composite entities can be any biomedical entity, such as a gene variation, drug effect, or disease treatment. For example, the gene entity *VKORC1* (a key entity) is used as a modifier of *expression* in *“warfarin inhibits the expression of VKORC1.”* Specifically, composite entities are composed of a sequence of terms that can be read left to right and where left term progressively specializes the term on its right. The last word is named the head entity. Figure 2 shows the components of relationships.

### Identification of a sentence with PGx relationships

Given the definition of PGx relationships, a sentence that potentially contains a PGx relationship would mention a gene and drug, a gene and a phenotype, or a drug and a phenotype. We used a Lucene index created on individual sentences of MEDLINE abstracts published before 2009 (17,396,436 abstracts and 87,806,828 sentences) processed by Xu *et al.* to identify those sentences that might contain a PGX relationship [12,13]. To select only sentences that potentially mention a PGx relationship we queried the index with pairs of key PGx entities (only gene-drug and gene-phenotype pairs) for sentences that are indexed with both the terms in the query. The PharmGKB lexicon, provides the sets of synonyms used to build such queries for the key entities. Overall, for this study we used 41 genes highlighted by PharmGKB as key, well characterized pharmacogenomic genes [14], as well as 3,007 drugs and 4,202 phenotypes. Future work will expand the relationship extraction to all genes.

### Extraction of heterogeneous raw relationships

Sentences returned by the index are parsed using the Stanford Parser to build Dependency Graphs (DGs) [15]. DGs are rooted, directed, and labelled graphs, where nodes are words and edges are dependency relations between words (*e.g.*, noun modifier, nominal subject). The extraction of raw relationships of the form R(*a*,*b*) relies on the exploration of syntactic structure provided by DGs where:

*- a* and *b* are nodes or chains of nodes in a DG, depending on whether they are a single key entity (an instance of gene, drug or phenotype) or a composite entity;

- R is a node in the DG that connects *a* and *b*, and indicates the nature of their relationship.

We have developed an algorithm to explore the DG and extract raw relationships from the raw text. The extraction of raw relationships is constrained by a set of rules defined using the different type of dependencies that associate nodes in DG. This step results in the extraction of over 40,000 raw relationships discussed in [11]. These relationships are highly heterogeneous and contain multiple equivalent ways to express one single fact. The details of the DG exploration algorithm appear in Table 1 of [11].

**Table 1:** Normalization algorithm

Algorithm for the normalization of composite entities using a domain ontology

### Building the PHArmacogenomic RElationship ontology

**Figure 3:**
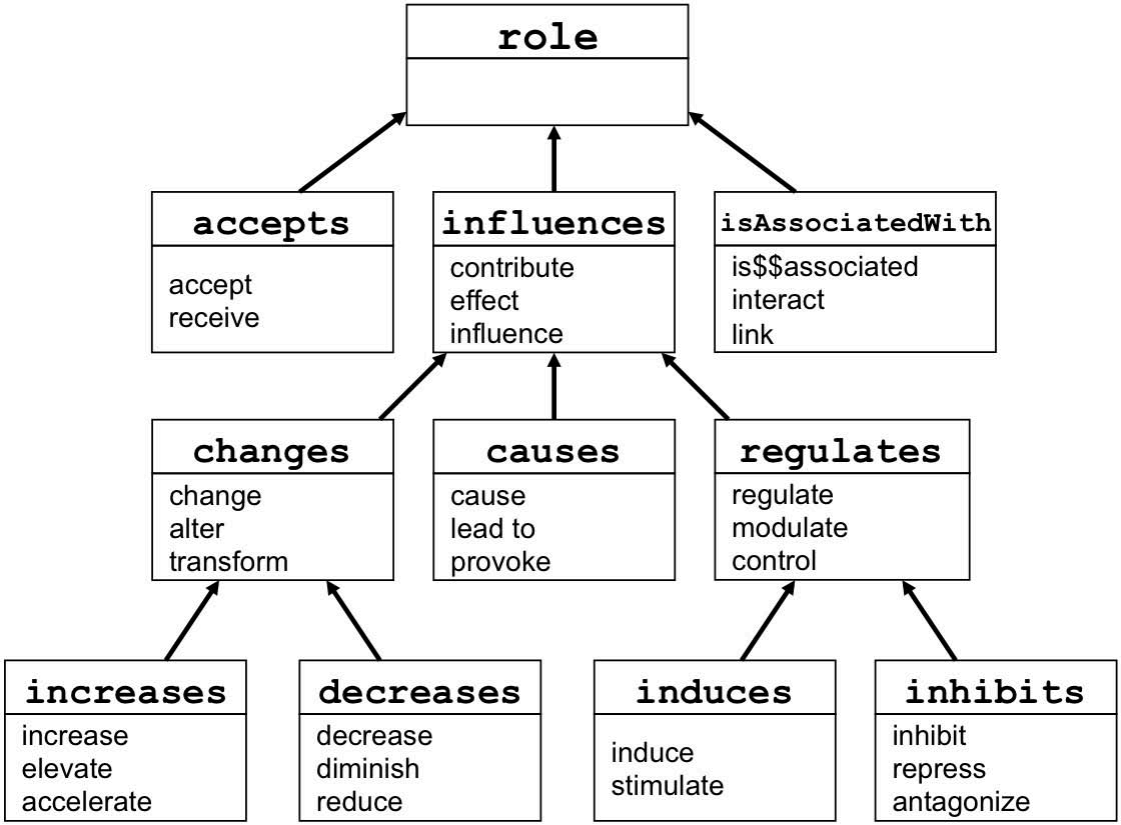
**A portion of the role hierarchy of the PHARE ontology.** Each box represents a role and words in the lower part of the box are the alternative labels for that role. Arrows represent sub-role relation. Each label can only belong to one role.

In order to create a smaller, normalized set of relationships, we first identified the 200 most frequent relationship types from the ~40,000 raw relationships. In the next step, we manually merged similar relationships and organized them hierarchically. Groups of similar relationships are used to define roles in the PHARE ontology. For example Figure 3 shows how *inhibit*, *repress*, and *antagonize* are merged to define the role inhibits. Role labels are declared using the rdfs:label annotation property. The first label of each role is used as its preferred name. Please note that the symbol $$ is a simple separator symbol that enables us to distinguish the passive voice from the simple past during the next normalization step.

**Figure 4:**
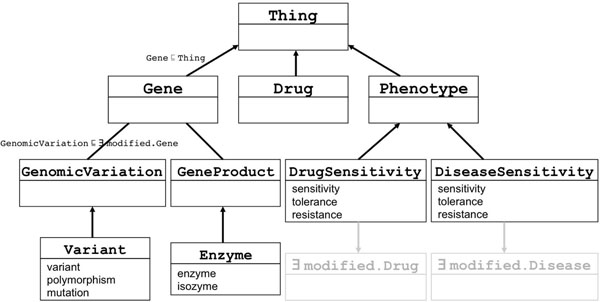
**A portion of the Entity hierarchy of the PHARE ontology**. Each box represents an entity type and terms in the lower part of the box are the alternative labels for that entity. Subsumption relations are represented with arrows. Non-hierarchical relations are represented without arrow.

In a similar manner we identified the 200 most frequent terms modified by key entities (e.g., *expression* for gene names or *sensitivity* for drug names). Then five PGx experts, including 3 co-authors and 2 PharmGKB curators, manually merged similar ones and organized them hierarchically in the entity hierarchy. Figure 4 shows how *variant*, *polymorphism*, and *mutation* are merged to define the entity Variant.

The entity hierarchy is defined with the subsumption relation (noted as ⊑ or subClassOf in OWL). Existential quantification is used to define sets of composite entities that are only modified by certain concepts. For example the set of entities that are modified by drugs is defined with the existential quantifier (Ǝ) and the role modified by: Ǝ modified.Drug (or modified someValuesFrom Drug in Manchester OWL syntax), see Figure 4 for examples. This definition is associated through a subsumption relation to entities that can be modified by drugs, such as DrugSensitivity. This pattern is used to distinguish what thing is specialized (or modified) by drugs from what is specialized by other modifiers (e.g. disease names). For example *warfarin* that we know to be a drug enables us to distinguish *warfarin sensitivity* from *cancer sensitivity* and to classify *warfarin sensitivity* as a kind of drug sensitivity versus disease sensitivity (represented by the DiseaseSensitivity concept).

**Figure 5:**
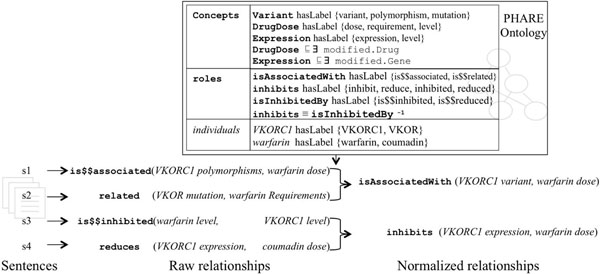
**Integration of heterogeneous relationships**. Four raw relationships are normalized to two expressions, using the PHARE ontology. The first two (s1 and s2) mention the same relationships with different words and sentence structures and are consequently integrated (e.g. ‘drug dose’ and ‘drug requirement’ are declared synonyms). s3 illustrates the utility of being able to distinguish between concepts modified by Gene and by Drug to disambiguate two different occurrences of “level”: one specialized by a gene name, the other by a drug name. Given the ontology, ‘gene level’ is a reference to gene expression, whereas ‘drug level’ refers to drug dose. s3 and s4 illustrate the utility of role inverses in the ontology, which enable the integration of relationships extracted from s3 and s4 by swapping subject and object of s3. The last two raw relationships are inverses that express the same relationship.

Inverse roles are explicitly defined using the inverse constructor (-1 or inverseOf in OWL). As shown in the example in Figure 5, roles inhibits and isInhibitedBy are inverses of one another.

Class declarations are used to list all key entities of the domain of interest and what entity type they belong to. In our case, where gene-drug relationships are studied, known drugs and genes must be defined in the ontology as being an instance of the entity types Drug and Gene.

### Building of WN-PHARE ontology using WordNet

In order to quantify the utility of manual review and editing of the raw relationships in building PHARE, we built a second ontology named WN-PHARE in a purely automated manner using the lexical resource WordNet [16]. In this case all relationship types—and not just the 200 most frequent ones—are computationally merged in groups according to WordNet synsets. Resulting groups are directly used to define roles without any manual review. Similarly, all terms that modify gene, drug or phenotype names are merged in groups used to define composite entities.

### Normalization and integration of heterogeneous relationships

The algorithm to normalize typed relationships between composite entities consists of four steps. The first three steps normalize the subject entity, the object entity, and the relationship type. The last step, assembles the three normalized pieces in a normalized relationship of the kind shown in Figure 1.

#### Normalization of composite entities (steps 1 and 2)

**Figure 6:**
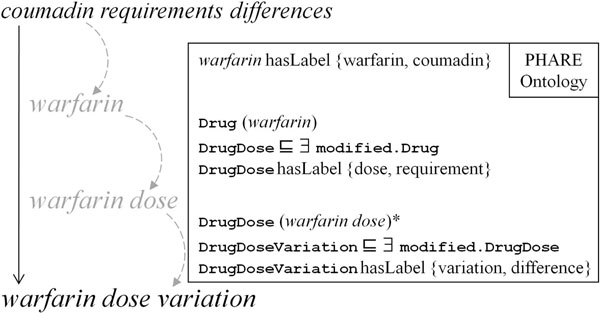
**Normalization of a composite entity.** Starting with the text *“differences in coumadin requirements”*, NLP tools generate the raw entity “*coumadin requirements differences*” on which we can apply the normalization algorithm (described in table 1) using the PHARE ontology. The first step ensures that the preferred name *warfarin* is used instead of coumadin. The second step maps “requirements” to the entity type DrugDose, and the final step maps “differences” to the entity type Variation. The axiom noted with a * is added to the ontology during the normalization as a result of the inference that a variation in drug dose was found.

This step—described in Table 1—takes as input a raw composite (or atomic) entity and the PHARE ontology to return a normalized entity. The first word of the entity is recognized as the key entity. Then each following word that composes the entity is considered from left to right as something further specialized by previous words. The ontology is searched for an entity label that matches with the processed word (named read_word in the Table 1 algorithm). This algorithm is applied successively to the subject entity and the object entity of a relationship (Figure 6).

#### Normalization of relationship types (step 3)

The next step is to normalize the relationship type. The ontology is searched for role labels that match the raw relationship. When a match is found, the preferred name of the corresponding role is used to normalize the relationship type. Note that during this step the normalization process distinguishes between passive voice of the present tense, such as “*A is inhibited by B*” and active voice of simple past tense “*B inhibited A*”. Dependency Graphs of these two sentences are different because “*inhibited*” in the passive voice sentence is related through an *aux* dependency to “*is*” (standing for auxiliary). This difference is used during the relationship extraction to extract either is$$inhibited(A, B) or inhibited(A, B).

#### Assembly of normalized pieces (step 4)

The final step is to group together normalized composite entities and relationship type to produce normalized relationships. For each relationship, this step relies on the simple assembly of normalized type, subject and object. In addition if the role used to normalize the type has inverses or is symmetric then this step also creates the appropriate additional relationships. For each inverse role in the ontology, an inverse relationship is created with the preferred name of the inverse and where normalized subject and object are swapped. If the role is symmetric, one additional relationship is created with the same normalized relationship type but with subject and object swapped. Figure 5 illustrates the integration process that applies such relationship normalization on four heterogeneous sentences.

Applying the normalization on raw relationships produces a set of relationships represented as PHARE entities and roles. Consequently normalized relationships can be directly added to PHARE as instances to create a knowledge base.

#### Refinement of PHARE by repeating the normalization step

Raw relationships have been normalized twice using PHARE to iteratively refine the ontology. After the first iteration of the normalization, from the pool of un-normalized relationships we manually identify terms and roles that are either frequent or of PGx interest. Such terms (or roles) are then used to extend the set of synonyms of an entity already defined in the ontology, or used to create a new entity in the ontology.

### Visualizing gene-disease networks

**Figure 7:**
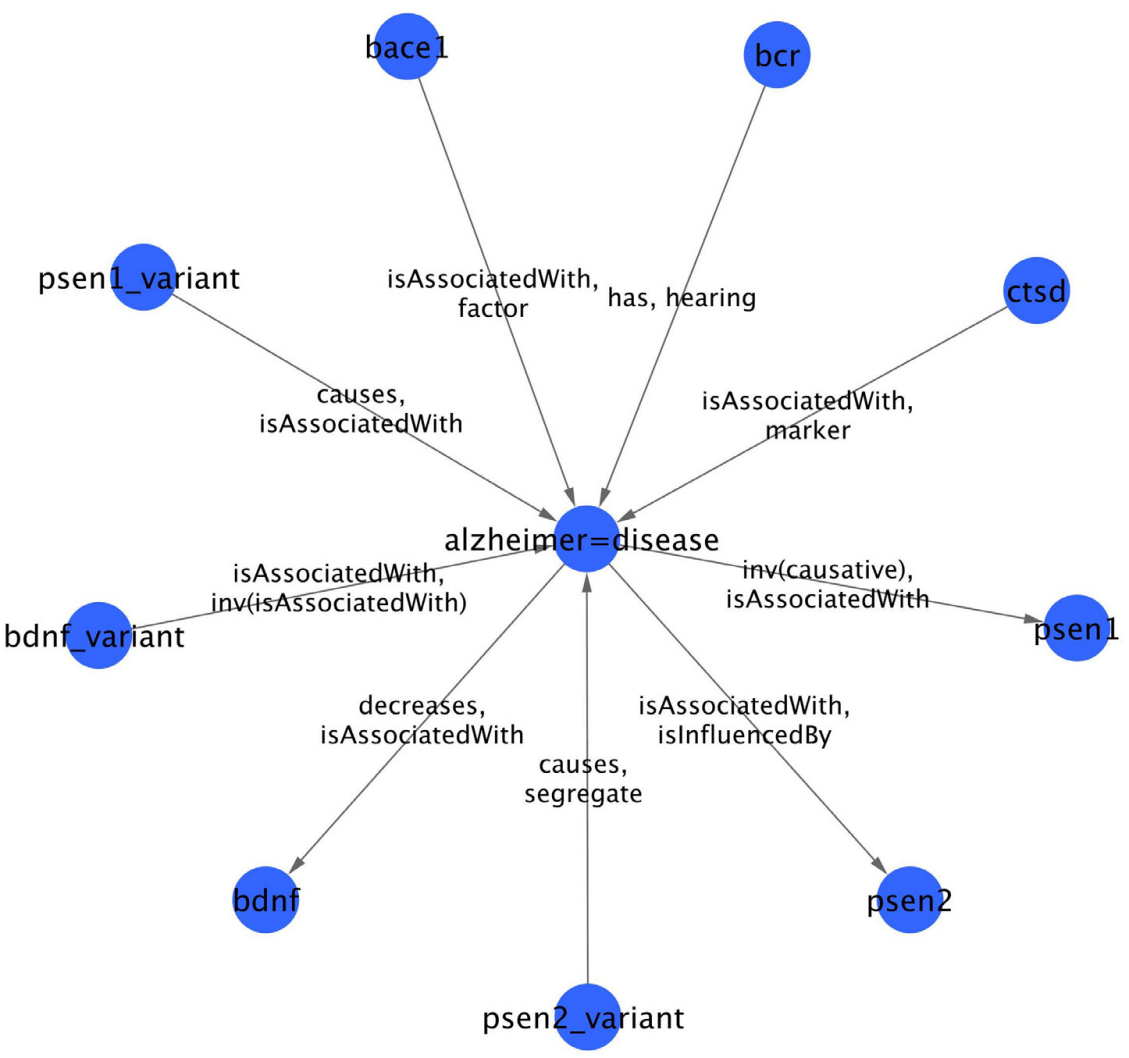
**Sub-network related to Alzheimer's disease**. Sub-network of genes (or associated entities) strongly related to Alzheimer's Disease (AD) according to PHARE-KB. Linked entities are linked by more than 5 sentences in MEDLINE abstracts. Relationships shown on the edges are the two most frequent type of relations mentioned in these sentences. Some relationships type are false such as “hearing”.

**Figure 8:**
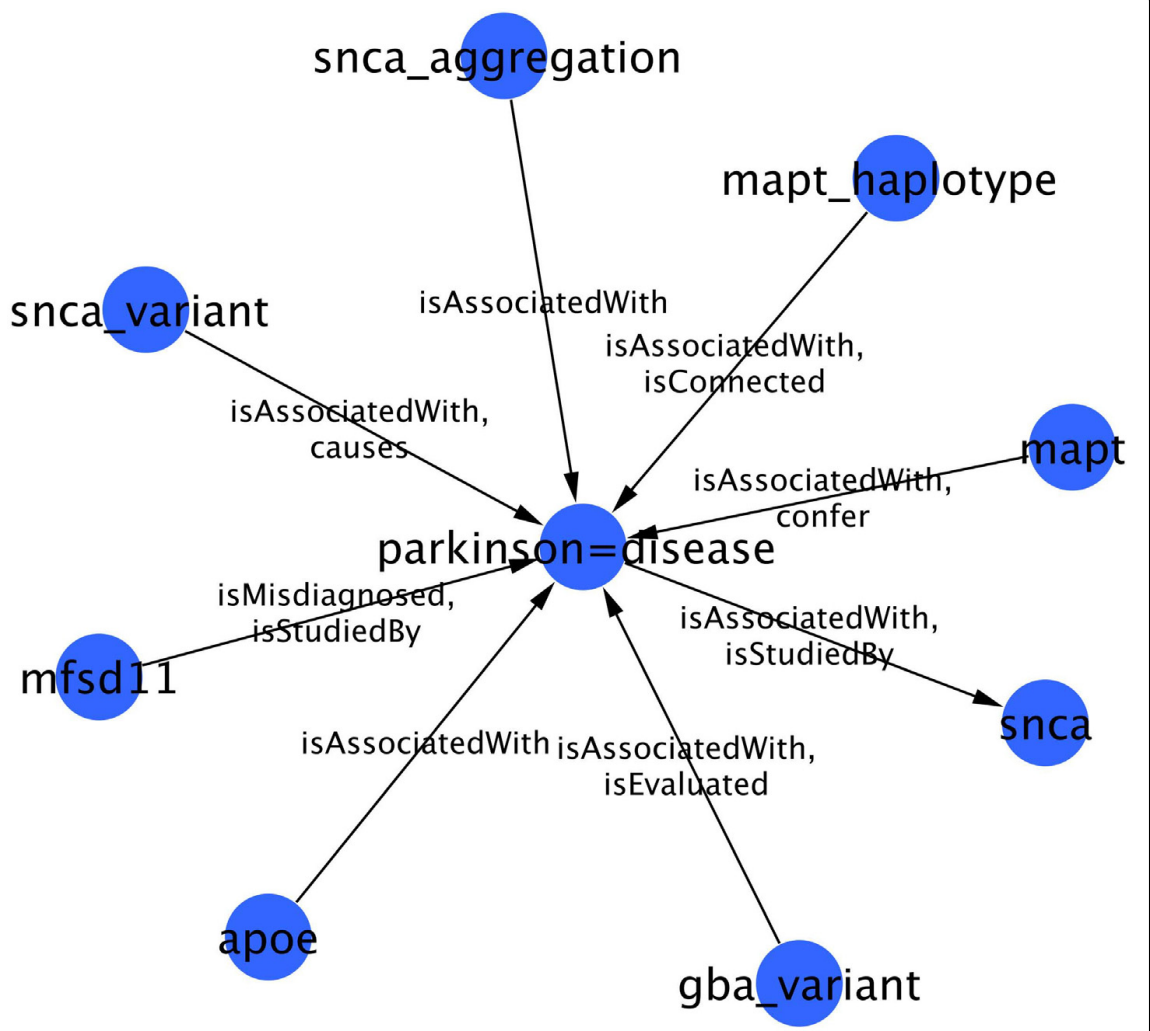
**Sub-network related to Parkinson's disease.** Sub-network of genes (or associated entities) strongly related to Parkinson's Disease (PD) according to PHARE-KB. Linked entities are linked by more than 5 sentences in MEDLINE abstracts. Relationships shown on the edges are the two most frequent type of relations mentioned in these sentences.

Figures 7 and 8 are made using a RDF to GML (Geography Markup Language) converter developed in-house. This converter enables the representation of RDF graphs in GML. GML files are then visualized and edited using Cytoscape 2.7 [17].

## Results

### The PHARE ontology

The PHArmacogenomic RElationship ontology (or PHARE) contains 229 entity classes and 76 roles of interest in the PGx domain. PHARE is encoded in OWL-DL and is constructed semi automatically by (*i*) listing terms derived from relationships extracted automatically from text ; and (*ii*) the manual organization of the relationship terms by domain experts. Figures 2 and 3 illustrate how the extracted terms are organized in these hierarchies. The PHARE ontology is available online at http://purl.bioontology.org/ontology/PHARE.

### The PHARE-Knowledge Base (PHARE-KB)

The ontology-driven integration process described in the method section takes as input a set of relationships extracted from MEDLINE abstracts and outputs a set of normalized relationships of the form Role(*subject*, *object*) represented using entity types and roles defined in PHARE. Therefore, normalized relationships can be used to instantiate roles defined in PHARE without additional processing. We performed such instantiation and obtained the PHARE-Knowledge Base (or PHARE-KB) that contains 28,676 roles instantiations encoded as RDF triples from over 41,000 raw relationships. If we consider instantiation of role inverses (e.g., isInhibitedBy (*a*,*b*) ≡ inhibits^-1^ (*b*,*a*)), the number of role instantiations rises to 46,526. Note that some roles in PHARE do not have inverse or are symmetric (e.g., isAssociatedWith).

Almost 77% role instantiations use roles initially encoded in PHARE and 23% necessitate the creation of new roles in PHARE. In other words PHARE roles are sufficiently detailed to capture 77% of the relationships we extracted from text analysis. New roles correspond to types of relationships that are not frequent enough in our corpus and consequently have not yet been manually reviewed and defined in PHARE. These roles, which are added solely to instantiate the 23% of un-normalized relationships are associated with only one, label and thus do not yet contribute to the integration of relationships.

The 28,676 role instances link roughly 16,000 individuals of the KB, including 285 genes, 1,083 drugs and 990 diseases. To facilitate overlap comparisons of PHARE-KB with other data sources individuals that are of type genes, drugs, or diseases are associated with their Entrez Gene, DrugBank, and MeSH identifiers respectively.

Individuals in the PHARE-KB can be classified using reasoning. Classification allows us to make the implicit knowledge units explicit. For example, classification infers that

Phenotype(*VKORC1 expression*)

i.e., VKORC1 expression is a phenotype

on the basis of the following two axioms

Expression(*VKORC1 expression*)

Expression ⊑ Phenotype

i.e., *VKORC1 expression* is a gene expression and gene expression is a phenotype.

Every relationship available in the PHARE-KB (in the form of a RDF triple) is associated with its provenance using the property rdfs:comment. For example, the triple isAssociatedWith(*UCHL1*, *parkinson disease*) is associated with the following string: ”[14522054, Neuronal ubiquitin C-terminal hydrolase (UCH-L1) has been linked to Parkinson's disease (PD), the progression of certain nonneuronal tumors, and neuropathic pain]”, Where 14522054 is the PMID (PubMed ID) of the article and the text is the sentence based on which the triple is created.

### Evaluation and comparison

To evaluate the impact of the manual review and curation in the construction of the PHARE ontology, we constructed an alternate relationship ontology—named WN-PHARE—in a fully automated manner using WordNet as described in the methods section. Table 2 compares the structure and the effectiveness of PHARE and WN-PHARE in integrating heterogeneous text-mined relationships. These features are measured for the task of integrating a subset of relationships extracted for Parkinson's Disease (PD). This subset contains 2,827 PD relationships extracted from 2,124 distinct MEDLINE abstracts. Logic criteria (e.g., satisfiability) of the ontologies are not included in the comparison since both ontologies are consistent and coherent.

**Table 2:** Comparison of PHARE and WN-PHARE

*Ontology*	*Number of entity types*	*Number of roles*	*Labels per entity type*	*Labels per role*	*Reduction*	*Coverage*
PHARE	229	77	3.91	6.06	64%	77%
WN-PHARE	1327	591	2.18	3.38	31%	89%

Comparison of PHARE (built semi automatically with added manual review and curation) and WN-PHARE (built in a fully automated manner). The *Reduction* column quantifies the ability of each ontology to normalize text-mined relationships. *Reduction* is the ratio of the number of normalized relationships and the initial number of raw relationships. The *Coverage* column quantifies the fraction of raw relationships that are normalized using roles and entity types encoded in the ontology.

We find that the roles represented in PHARE cover the set of extracted relationships incompletely but they normalize more relationships than the roles in defined in WN-PHARE. Thus the manually reviewed ontology results in a better identification of similar relationships that are phrased differently in natural language, but it captures a smaller fraction of the total relationships extracted from text. Table 3 provides additional evaluation with numbers of similar relationships (same subject, predicate and object) identified first before normalization, second after normalization using PHARE, and third after normalization using WN-PHARE.

**Table 3:** Comparison of the identification of similar relationships

		*Raw relationships (no normalization)*	*Relationships normalized with*	
			*PHARE*	*WN-PHARE*
*Number of relationships identified **n** times*	*2* ≤ ***n*** <*5*	7	87	70
	*5* ≤ ***n*** <*10*	0	12	6
	***n*** ≥ *10*	0	5	2

Comparison of the occurrence of relationships in three differentially normalized sets of relationships. Identifications are made on 2,827 relationships related to Parkinson’s Disease. Before any normalization only 7 distinct relationships can be identified as occurring several times. Normalization with PHARE and WN-PHARE (built semi automatically) and WN-PHARE (built in a automated manner) enable to reveal more identical relationships. For instance, with PHARE normalization, 5 relationships are found to occur more than 10 times (*n* ≥ *10*).

### SPARQL query point

In order to publish the PHARE-KB for use on the Semantic Web, we set up a SPARQL endpoint, which is available at http://sparql.bioontology.org/webui/. Examples of queries are provided as additional file 1.

The KB is classified and inferred triples are materialized before loading into the triple store underlying the SPARQL endpoint. As a consequence queries return asserted as well as inferred facts.

An example of query for entities related to the uchl1 gene is shown below:

SELECT $y $z

FROM <http://www.stanford.edu/~coulet/phare.owl>

WHERE <http://www.stanford.edu/~coulet/phare.owl#uchl1> $y $z;

This query returns the RDF triple isAssociatedWith(*UCHL1*, *parkinson disease*) mentioned previously. Queries can also return sets of RDF triples that are used to build sub-network related to a specific diseases as shown in Figure 7.

### Disease related gene networks

Figures 7 and 8 show gene-disease sub-networks related to AD and PD respectively. For display purpose, these have been reduced by selecting only those nodes that are asserted to be related in more than 5 different sentences. Since the type of relationship differ in sentences, only the two most frequent relationships are displayed as labels on the edges. Each network was obtained using a SPARQL query to select triples where the disease (AD or PD) is either subject or object. Resulting set of triples is then filtered to keep the frequent relationships. Such filtering enables to us remove both false positives as well as irrelevant triples such as phare:alzheimer=disease rdf:type phare:Disease . Note that in RDF we use the symbol ‘=’ as a simple separator to replace spaces in coumpound nouns.

## Discussion

Our work is motivated by the need for automated approaches capturing and formalizing knowledge extracted from the literature and the need for publishing such knowledge on the Semantic Web. Recent advances in Natural Language Processing (NLP) techniques enable the extraction of fine-grained relationships mentioned in biomedical text [4]. The variability and the complexity of natural language in expressing similar or simple relationships causes the extracted relationships to be highly heterogeneous. We show that the use of a relationship ontology can normalize and integrate the heterogeneous relationships extracted from text and serve as a common semantic framework to integrate text-mining derived facts into a knowledge base. However, the manual construction of a relationship ontology is a slow and expensive process [18]. We have devised a method to construct such an ontology using the text-extracted heterogeneous relationships as a starting point. Although we only report on our experiments in the pharmacogenomics domain; we note that the approach described here can be applied for relationship extraction in other domains.

### Linked data cloud and text-mined relationships

Our results in publishing RDF triples extracted from text align closely with the objectives of the Linking Open Data community project [19] and that of efforts such as the Concept Web Alliance [20]. The goal of projects such as Linked Open Data is to publish various data sets as RDF on the Web and to declare links between data items from different data sources.

Currently, the relationships we extract do not integrate easily with content in the Link Data Cloud for two main reasons: the lack of resource unique identifiers and the lack of an agreed upon relation ontology. Despite community efforts to create unique resource identifiers for life sciences, currently there is no clear consensus [21,22]. In addition, composite entities, such as *VKORC1 expression* that participate in relationships are too complex to reference using a single identifier. Moreover, the absence of an expressive and comprehensive relation ontology led us to develop our own in a boot-strapped manner from example instances of text-mined relationships. PHARE is designed for the purpose of representing PGx relationships and we anticipate that sharing it with the community will provide a much needed example set for the development of a proper, formal biomedical relation ontology. PHARE is particularly suited to seed that activity, because it is built from the most frequent relationships that are used in the scientific literature. One challenge is thus to propose consistent mappings between relationship types arising from the literature, such as those suggested by PHARE and relationship types arising from functional annotations such as “suppresses gene” or “enhances gene” suggested by TAIR relations or the Gene Ontology [23].

### Limitations of our approach

Adequately representing provenance information at the sentence level is a challenge. Currently, we utilize the rdfs:comment property to store provenance for each extracted fact in PHARE-KB. In the future, we plan to evaluate the Annotation Ontology developed by Ciccarese et al. [24] for its utility is representing provenance at the sentence level, particularly in workflows where both automated and manual approaches are used simultaneously.

Another limitation is the incoherence between gene name identifiers across data sources. Our gene identifiers are based on PharmGKB gene names that are not entirely consistent with the HUGO Gene nomenclature [25], making cross referencing with other sources time consuming. In a similar vein, recall for extracted relations may improve upon using advanced Named Entity Recognition such as disambiguation techniques rather than the current PharmGKB-derived dictionary based approach.

The efficacy of the relationship normalization and integration might vary depending on the source of the text such as full articles, clinical reports, clinical files or drug labels. However, because PHARE has been designed using MEDLINE abstracts, it may capture relationships mentioned in diverse sources.

## Conclusions

We have described the construction of an ontology of relationships in the PGx domain and its use to integrate heterogeneous relationships extracted by text-mining. The synonyms, entity descriptions, and the hierarchies of entities and roles represented in the ontology are used to map text-derived relationships to the ontology. Once mapped, relationships can be normalized and compared using the semantics defined in the ontology to identify relationships that have similar semantics but different syntax. We compare and contrast a fully automated and a manually edited version of the PHARE ontology to quantify the degree of integration enabled by manual inspection, curation and refinement of the PHARE ontology. PHARE has been successfully used in a pipeline for the integration of pharmacogenomic relationships extracted from MEDLINE abstracts [11]. The result of the integration is compiled into a knowledge base named PHARE-KB, which can now be queried using Semantic Web technologies such as SPARQL and can be visualized in the form of a biological network. PHARE-KB can also be queried programmatically, for example, to guide computational prediction of molecular interactions [26].

## List of abbreviations used

AD: Alzheimer's Disease; DG: Dependency Graph; KB: Knowledge Base; NER: Named Entity Recognition; NLP: Natural language Processing; OWL: Web Ontology Language; OWL-DL: Web Ontology Language, Description Logic Kind; PD: Parkinson's Disease; PHARE: PHArmacogenomic Relationships; PGx: Pharmacogenomics; RDF: Resource Description Framework; SPARQL: SPARQL Protocol And RDF Query Language.

## Competing interests

Authors declare no competing interests.

## Authors' contributions

AC designed and implemented the ontology driven relationship normalization as well as wrote the manuscript. YG improved the approach and the ontology. MD contributed to the evaluation and discussion sections. YG and MD critically reviewed the manuscript. RBA and MAM obtained funds and gave scientific directions. NHS supervised the project, participated in technical discussions and wrote the manuscript.

## Supplementary Material

Additional file 1 Examples of SPARQL queries Description of data: This file proposes examples of SPARQL queries that can be used to query PHARE-KB on the SPARQL endpoint set up at http://sparql.bioontology.org/webui/ Click here for file
